# Mechanisms underlying age-associated exacerbation of pulmonary veno-occlusive disease

**DOI:** 10.1172/jci.insight.181877

**Published:** 2024-09-05

**Authors:** Amit Prabhakar, Meetu Wadhwa, Rahul Kumar, Prajakta Ghatpande, Aneta Gandjeva, Rubin M. Tuder, Brian B. Graham, Giorgio Lagna, Akiko Hata

**Affiliations:** 1 Cardiovascular Research Institute,; 2 Department of Anesthesia and Perioperative Care, and; 3 Department of Radiology, UCSF, San Francisco, California, USA.; 4 Lung Biology Center, Pulmonary and Critical Care Medicine, Zuckerberg San Francisco General Hospital, California, USA.; 5 Department of Medicine, University of Colorado School of Medicine, Aurora, Colorado, USA.; 6 Department of Biochemistry and Biophysics, University of California, San Francisco, San Francisco, California, USA.

**Keywords:** Therapeutics, Vascular biology, Cardiovascular disease, Cell stress, Hypertension

## Abstract

Pulmonary veno-occlusive disease (PVOD) is a rare but severe form of pulmonary hypertension characterized by the obstruction of pulmonary arteries and veins, causing increased pulmonary artery pressure and leading to right ventricular (RV) heart failure. PVOD is often resistant to conventional pulmonary arterial hypertension (PAH) treatments and has a poor prognosis, with a median survival time of 2–3 years after diagnosis. We previously showed that the administration of a chemotherapy agent mitomycin C (MMC) in rats mediates PVOD through the activation of the eukaryotic initiation factor 2 (eIF2) kinase protein kinase R (PKR) and the integrated stress response (ISR), resulting in the impairment of vascular endothelial junctional structure and barrier function. Here, we demonstrate that aged rats over 1 year exhibit more severe vascular remodeling and RV hypertrophy than young adult rats following MMC treatment. This is attributed to an age-associated elevation of basal ISR activity and depletion of protein phosphatase 1, leading to prolonged eIF2 phosphorylation and sustained ISR activation. Pharmacological blockade of PKR or ISR mitigates PVOD phenotypes in both age groups, suggesting that targeting the PKR/ISR axis could be a potential therapeutic strategy for PVOD.

## Abstract 2

Pulmonary veno-occlusive disease (PVOD) is a form of pulmonary hypertension (PH) with more severe vascular remodeling in old rats which can be mitigated by targeting the PKR-ISR pathway.

## Graphical abstract

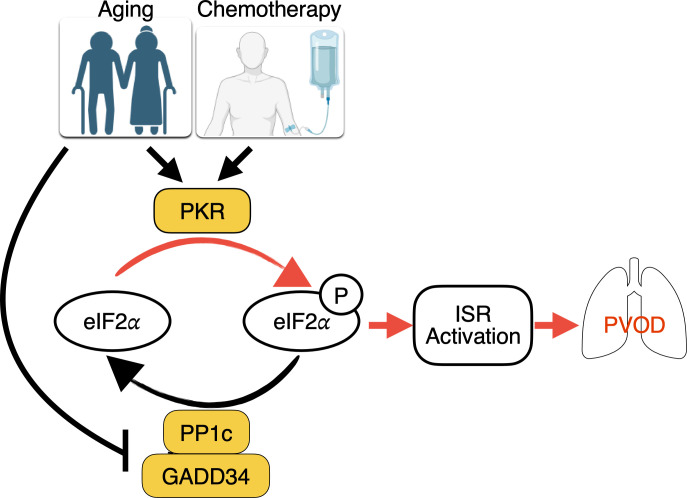

## Introduction

Pulmonary veno-occlusive disease (PVOD), or pulmonary capillary hemangiomatosis, is a subclass of pulmonary hypertension (PH) affecting males and females at a prevalence of 1–2 cases per 10 million population (1). PVOD is characterized by the progressive remodeling and obstruction of small pulmonary arteries (PAs), pulmonary veins (PVs), and pulmonary capillaries (PCs), leading to increased PA pressure and RV failure (2, 3). PVOD can begin at any age, with the average onset occurring between 30 and 50 years old (2). Currently, there is no effective therapy for PVOD, and the mortality rate is 72% within 1 year of diagnosis (2). Vascular lesions in patients with PVOD also include thrombosis, fibrous intimal proliferation, and dilatation and proliferation of PCs (2). Biallelic loss-of-function/expression mutations in the *Eif2ak4* gene, encoding the general control nonderepressible 2 (GCN2), are the primary genetic cause of PVOD (4, 5). PVOD is often misdiagnosed as pulmonary arterial hypertension (PAH) due to similarities in radiographic findings and the overlap of gene mutations between PVOD and PAH (4, 5). Patients with PVOD frequently develop life-threatening complications after receiving vasodilator therapies for PAH, highlighting the critical need for therapeutics specifically targeting PVOD (2). Pharmacologically, the administration of alkylating agents, such as mitomycin C (MMC), bleomycin, and cisplatin, has been implicated in the onset of PVOD (6–8). Specifically, MMC administration in rats induces a spectrum of PVOD-like phenotypes, including RV hypertrophy and changes in the pulmonary vasculature, such as medial thickening and obstruction of the lumen in PAs and PVs, fibrous growth in the intima and adventitia, and thrombosis (7, 9–12). Therefore, MMC is the causal factor associated with PVOD. Furthermore, the MMC-mediated rat PVOD model offers valuable insights into the pathogenesis of the disease and serves as a platform for testing potential therapeutic interventions.

We demonstrate that when rats are administered with MMC, it instigates the integrated stress response (ISR) activation and impairs endothelial barrier function by depleting endothelial-specific adhesion molecule vascular endothelial–Cadherin (VE-Cad) (13) and Rad51, which interacts with VE-Cad and stabilizes its stability (12). Depleting the VE-Cad:Rad51 complex (VRC) from the adherens junctions (AJs) impairs endothelial cell-cell junctions, increases endothelial permeability, and subsequently triggers vascular remodeling that mimics the condition observed in patients with PVOD (12). The ISR is an evolutionary conserved adaptive intracellular mechanism that maintains homeostasis upon changes in the cellular environment and pathological stimuli (14). However, the activation of ISR is also associated with various disorders and age-related ailments in humans (14). The ISR is initiated by the activation of stress-responsive eIF2 kinases, which include HRI (EIF2AK1), protein kinase R (PKR) (EIF2AK2), PERK (EIF2AK3), and GCN2 (EIF2AK4) (14). Under stress conditions, eIF2 kinases become active and phosphorylate the α subunit of eIF2 (eIF2α) (15). When eIF2α is phosphorylated, cap-dependent translation is attenuated (14). However, the translation of some transcripts, including the main ISR effector, cyclic AMP-dependent transcription factor 4 (ATF4), is enhanced (14). As a result, ATF4-dependent transcriptional regulation of stress genes facilitates cellular adaptation and resilience (14, 16). When stress conditions are resolved, the ISR signal is attenuated by the dephosphorylation of eIF2α by protein phosphatase 1 (PP1), a crucial negative feedback regulation mechanism that helps restore protein synthesis upon stress resolution (14). PP1 consists of a catalytic subunit, PP1c, and either the stress-induced regulatory subunit growth arrest and DNA damage-inducible protein (GADD34, also known as PPP1R15A) or the constitutively expressed regulatory subunit constitutive repressor of eIF2α phosphorylation (CReP, also known as PPP1R15B) (14, 17). GADD34 is induced upon ISR activation through ATF4-dependent transcriptional activation, followed by a preferential translation of its transcripts (18–20). The role of the negative regulatory feedback mechanism for the ISR in PVOD pathogenesis has not been previously explored. Our previous study has demonstrated that MMC induces ISR activation via PKR. The simultaneous treatment with the PKR antagonist C16 or the ISR inhibitor ISRIB, alongside MMC, prevents the impairment of pulmonary endothelial cells and the development of PVOD phenotypes (12). In this study, we investigate the effects of aging and the therapeutic potential of PKR or ISR blockade in the rat model of PVOD. Our findings demonstrate that aged rats exhibit more severe PVOD phenotypes compared with young rats following MMC administration. MMC treatment diminishes PP1, leading to sustained eIF2α phosphorylation and prolonged ISR activation. The administration of PKR or ISR antagonists after the full development of PVOD successfully restores PP1, inhibits the ISR, and alleviates PVOD symptoms in both young and aged rats, suggesting that pharmacological inhibition of the PKR/ISR axis is an effective strategy for mitigating advanced PVOD, irrespective of the patient’s age.

## Results

### Increased ISR activity in aged rats.

**Figure 1:**
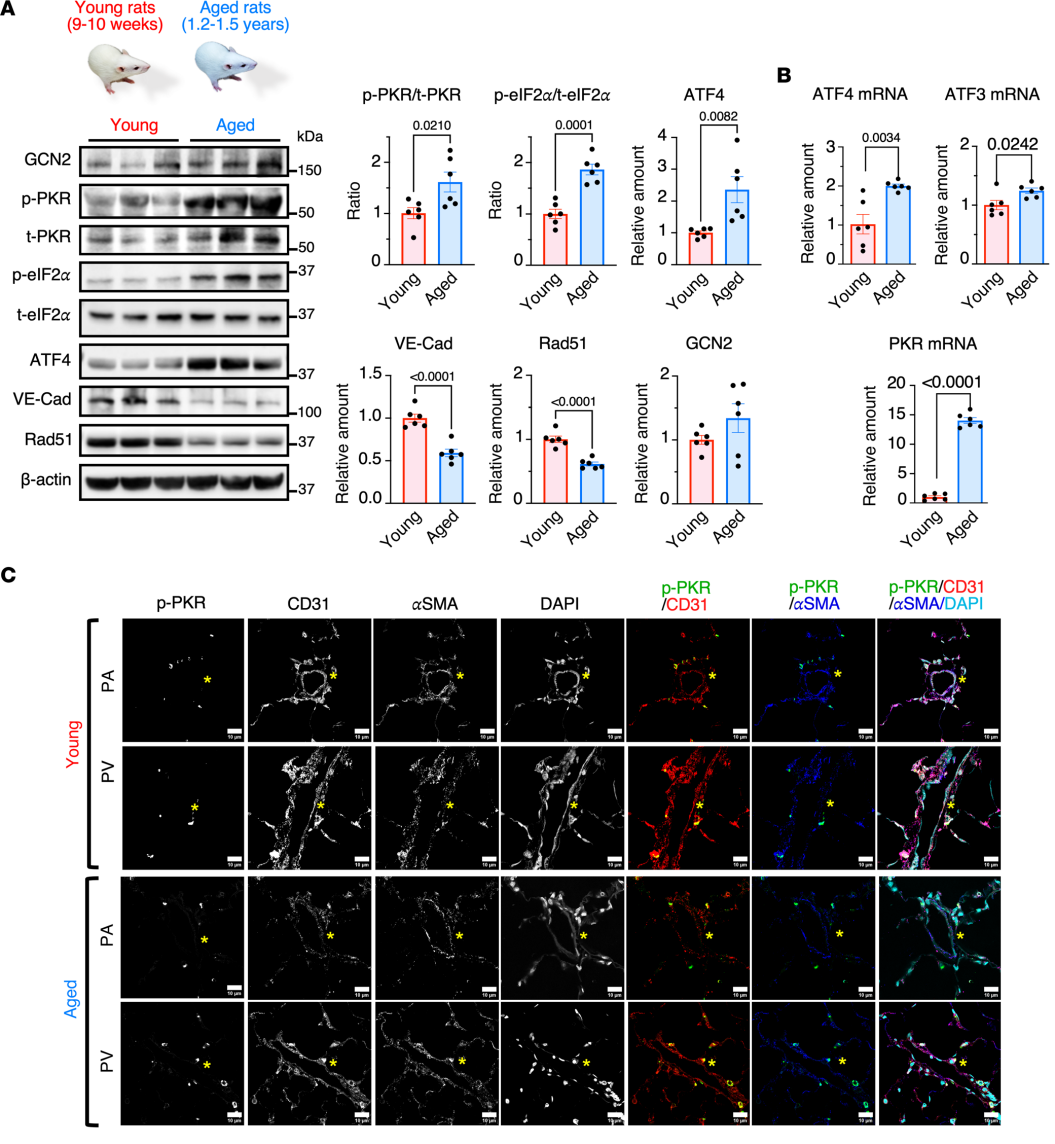
Age-dependent increase in the basal ISR activity. (**A**) Lung lysates of young and aged rats were subjected to immunoblot of the indicated proteins (left). The amounts of indicated proteins, normalized to β-actin, the ratio of p-PKR/t-PKR, and p-eIF2α/t-eIF2α are shown as mean ± SEM (right). *n* = 6 independent samples per condition. (**B**) The level of mRNAs of ATF4, ATF3, and PKR in the lung young and aged rats were analyzed by qPCR and shown as mean ± SEM after normalized to the GAPDH mRNA. *n* = 6 independent samples. (**C**) Lung samples from young and aged rats were subjected to IF staining with anti–p-PKR, anti-CD31, and anti-αSMA antibodies, and the images are shown. Cell nuclei were stained with DAPI. The asterisks indicate the location of the vessels. Scale bar: 10 μm. Statistical analysis was performed using a 2-tailed Student’s *t* test with *P* < 0.05.

Given that the median age of patients with PVOD at diagnosis is around 40, we hypothesized that an age-associated increase in ISR activity might contribute to the onset of PVOD in the fourth decade of life (2). The levels of ISR activity were compared between young adults (9–10 weeks old; equivalent to 19–21 years old in humans) and aged (1.2–1.5 years old; equivalent to 37–46 years old in humans) Sprague-Dawley rats (21). The levels of the active phosphorylated form of PKR (p-PKR) (2.7-fold), total PKR (t-PKR) (1.6-fold), the ratio of p-PKR to t-PKR (p-PKR/t-PKR) (1.6-fold), phosphorylated eIF2α (p-eIF2α) (1.8-fold), the ratio of p-eIF2α to t-eIF2α (p-eIF2α/t-eIF2α) (1.8-fold), and ATF4 (2.3-fold) (16) were elevated in aged rats compared with young rats.(Figure 1A and Supplemental Figure 1; supplemental material available online with this article; https://doi.org/10.1172/jci.insight.181877DS1). These results suggest the induction of basal ISR activity due to aging. In contrast, the levels of VE-Cad (13) and Rad51 (12) were 40% and 39% lower in aged rats than young rats, respectively (Figure 1A). There was no statistically significant change in the amount of GCN2 between young and aged rats (Figure 1A). The mRNA levels of ATF4 and ATF4 target genes, such as *ATF3* (22) and *PKR (Eif2ak2*) (12), were 2-fold, 1.2-fold, and 14-fold higher in aged rats than young rats (Figure 1B). Immunofluorescence (IF) staining of p-PKR in the lung sections detected a higher p-PKR signal compared with young rats in CD31^+^ vascular endothelial cells (vECs) but not in vascular smooth muscle cells (vSMCs) that are positive with the α-smooth muscle actin (αSMA) (Figure 1C). These results demonstrate that the basal activities of PKR and ISR increase with aging in the pulmonary vascular endothelium.

### More severe PVOD phenotypes in aged rats.

**Figure 2:**
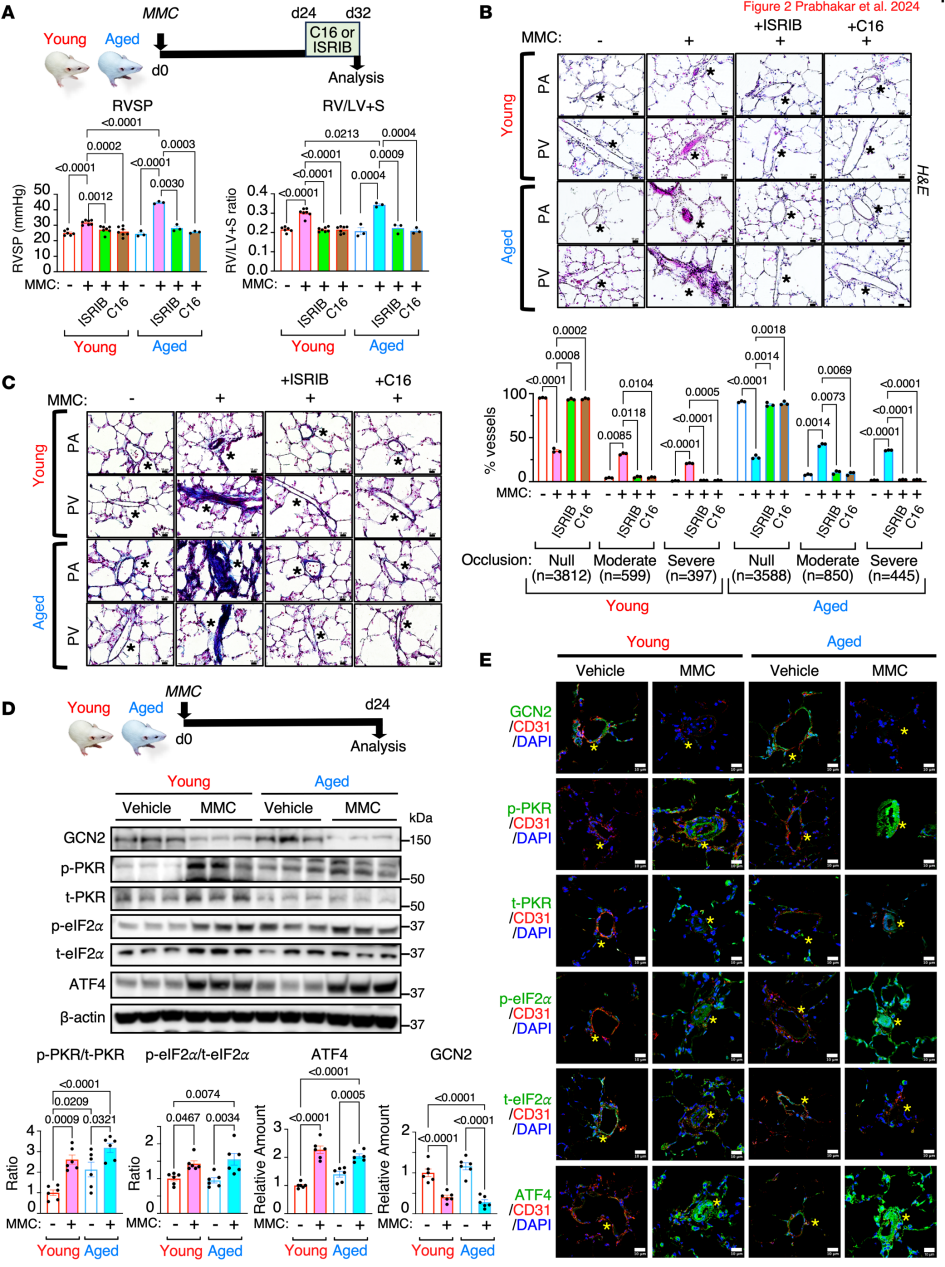
Administration of C16 or ISRIB 24 days after MMC treatment reverses PVOD phenotypes. (**A**) A scheme of delayed C16 and ISRIB treatment on d24–d32 after the administration of MMC on d0 in aged and young rats (top). The RVSP and the RV/LV+S ratio in vehicle- or MMC-exposed rats with or without C16 or ISRIB treatment are shown as mean ± SEM (bottom). *n* = 3–7 independent samples. (**B**) H&E staining of pulmonary vasculature (PAs and PVs) in young and aged rats treated with vehicle or MMC with or without C16 or ISRIB treatment was performed (top). A total number of microvessels was analyzed and distributed in 3 categories based on medial thickening/occlusion of pulmonary vasculature as null (no occlusion), moderate (25%–50% occlusion), and severe (50%–100%) in young (bottom, left) and aged (bottom, right) rats. The data are presented as a faction (%) of vessels in each category relative to the total vessels count and shown as mean ± SEM. Scale bar: 10 μm. *n* = 3 independent experiments. (**C**) Trichrome staining of pulmonary vasculature (PAs and PVs) in young and aged rats treated with vehicle or MMC with or without delayed C16 or ISRIB treatment was performed on d32 and shown as mean ± SEM. Scale bar: 10 μm. *n* = 3 independent experiments. (**D**) A scheme of MMC treatment in young and aged rats. Lung lysates of young and aged rats 24 days after vehicle or MMC administration were subjected to immunoblot of the indicated proteins. The amounts of indicated proteins, normalized to β-actin, the ratio of p-PKR/t-PKR, and p-eIF2α/t-eIF2α, are shown as mean ± SEM. *n* = 6 independent samples per condition. (**E**) Lung sections from young and aged rats administered with vehicle or MMC were subjected to IF staining using anti–p-PKR, anti–t-PKR, anti–p-eIF2α, anti–t-eIF2α, anti-GCN2, and anti-CD31 antibodies, and the images are shown. Cell nuclei were stained with DAPI. The asterisks indicate the location of the vessels. Scale bar: 10 μm. Statistical analysis was performed using 1-way ANOVA with Tukey’s multiple comparisons test (**A** and **D**) or 2-way ANOVA with Tukey’s multiple comparisons test (**B**) with *P* < 0.05.

We previously demonstrated that the simultaneous administration of MMC and the PKR antagonist C16 (23), or the ISR inhibitor ISRIB (24, 25), prevents the development of PVOD phenotypes, such as pulmonary vascular remodeling and an elevation of the RV systolic pressure (RVSP) and an increase in the ratio of the RV weight to the left ventricle (LV) + septum (S) weight (RV/LV+S) (12). Here, we tested the therapeutic effects of C16 or ISRIB by administering these agents after MMC-mediated PVOD phenotypes were established. Twenty-four days after the administration of MMC, young and aged rats were treated with vehicle, C16 (23), or ISRIB (24, 25) for 8 days, followed by the analysis of cardiovascular phenotypes (Figure 2A). The rats treated with MMC but not with C16 nor ISRIB (MMC/vehicle) exhibited the hallmarks of PH phenotype, such as elevation of PA pressure shown as RVSP (from 25.0 mmHg to 34.1 mmHg in young rats and from 24.4 mmHg to 34.6 mmHg in aged rats) and RV hypertrophy shown as the RV/LV+S ratio (from 0.21 to 0.31 in young rats and from 0.21 to 0.34 in aged rats) (Figure 2A). The aged rats demonstrated higher levels of RVSP and the RV/LV+S ratio than young rats (Figure 2A). We did not observe spontaneous development of PH in vehicle-treated aged rats (Figure 2A), despite an increase in basal ISR activity (Figure 1, A and B). When treated with either C16 or ISRIB, the RVSP and the RV/LV+S ratio were reversed to the levels similar to control (vehicle/vehicle-treated) young and aged rats (Figure 2A). H&E staining of the lung showed that both young and aged rats developed medial hyperplasia/hypertrophy in PAs and muscularization of PVs following MMC treatment (7, 9) (Figure 2B). There were no signs of spontaneous development of vascular remodeling in vehicle-treated aged rats (Figure 2B). This result is consistent with the lack of increase in the RVSP and the RV/LV+S ratio (Figure 2A). Both microvessels (<50 mm in diameter) and medium-sized vessels (50–80 mm in diameter) underwent medial thickening and intimal damage, resulting in the occlusion of vessels in both young and aged rats following MMC treatment (Figure 2B). The fraction of vessels with moderate (4.07%–31.56% increase in young; 7.78%–41.85% increase in aged rats) to severe (0.76%–20.67% increase in young; 1.46%–35.51% increase in aged rats) medial thickening elevated from 4.83% to 64.90% after MMC treatment in young rats and from 9.23% to 72.36% in aged rats, resulting in narrowing or complete obstruction of the lumen (Figure 2B). These results indicate that aged rats develop more severe vascular remodeling than young rats after MMC. When rats were treated with C16 or ISRIB, pulmonary vascular remodeling in both young and aged rats was reversed and indistinguishable from MMC-untreated rats (Figure 2B). Trichrome staining indicated that fibrous growth of intima and adventitia in young and aged rats after MMC, which was mitigated by C16 or ISRIB treatment in both young and aged rats (Figure 2C). The lung lysates of MMC-treated young and aged rats exhibited higher levels of in p-PKR/t-PKR ratio, p-eIF2α/t-eIF2α ratio, and ATF4 than untreated rats, demonstrating the ISR activation by MMC (Figure 2D). Although statistically not significant, the levels of ISR activity in aged rats were slightly higher than in young rats (Figure 2D). Similar to the immunoblot results of the lung lysates (Figure 2D), the IF staining demonstrated activation of the PKR/eIF2/ISR axis and a decrease in GCN2 in CD31^+^ vECs of both young and aged rats (Figure 2E). These results confirm that the activation of the PKR/eIF2/ISR axis in the vascular endothelium is causally linked to pulmonary vascular remodeling in PVOD.

### Reversal of PVOD phenotypes by blockade of the PKR/ISR axis.

**Figure 3:**
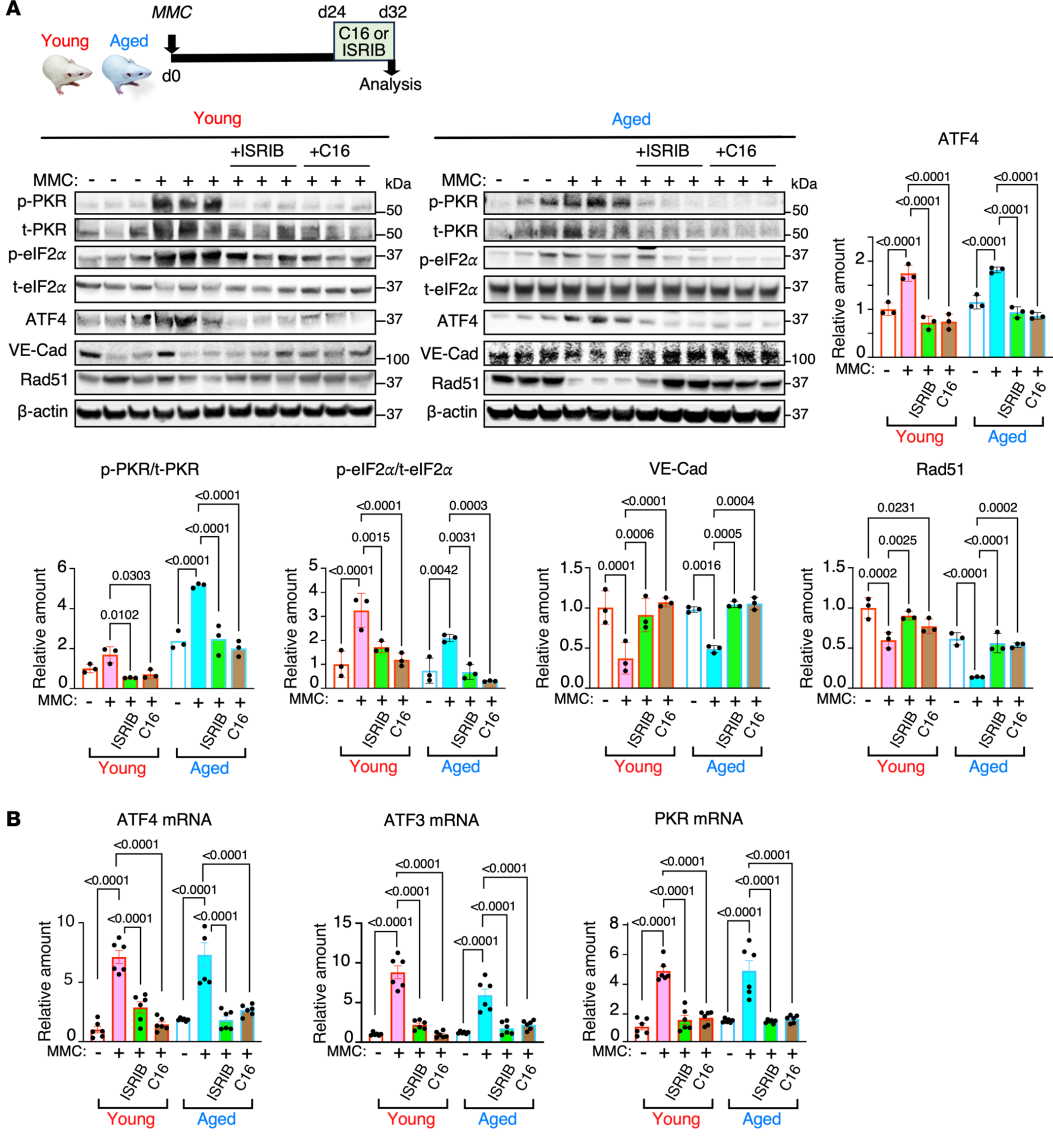
Delayed treatment with either PKR or ISR antagonists ameliorates MMC-mediated PVOD. (**A**) Immunoblot analysis of indicated proteins in total lung lysates from vehicle (–), MMC (+) with or without C16 or ISRIB in young and aged rats). The amounts of indicated proteins, normalized to β-actin, are shown as mean ± SEM. *n* = 3 independent samples. The relative quantity of proteins was calculated by setting the amount of the protein in the vehicle-treated young rat to 1. (**B**) The levels of mRNAs of ATF4 target genes, such as ATF4, ATF3, and PKR, in the lungs of young and aged rats administered with a vehicle, MMC, MMC+ISRIB, or MMC+C16 were analyzed by qPCR and shown as mean ± SEM after normalized to the GAPDH mRNA. *n* = 6 independent samples. The relative quantity of mRNAs was calculated by setting the amount of the mRNA in the vehicle-treated young rat to 1. Statistical analysis was performed using 2-way ANOVA (**A**) or 1-way ANOVA with Tukey’s multiple comparisons test (**B**) with *P* < 0.05.

To test the therapeutic potential of the PKR antagonist C16 and the ISR inhibitor ISRIB, young and aged rats were treated with these agents for 8 days, starting 24 days after MMC administration (Figure 3A). Delayed treatment with either C16 or ISRIB effectively reversed the increased levels of p-PKR/t-PKR, p-eIF2α/t-eIF2α, and ATF4, restoring them to basal levels (Figure 3A). Additionally, treatment with C16 or ISRIB restored VE-Cad and Rad51 levels to their basal states, suggesting a recovery of endothelial cell-cell adhesion and barrier integrity (Figure 3A). Moreover, the transcriptional activation of ATF4 target genes (*ATF3, ATF4,* and *PKR*) after MMC treatment was reversed by either C16 or ISRIB treatment in young and aged rats, confirming the inhibition of the PKR/ISR axis by C16 and ISRIB (Figure 3B). These results confirm that pharmacological intervention of the PKR/ISR axis by C16 or ISRIB can reverse MMC-mediated PVOD phenotypes even after fully developing them.

### Reduction of the intracellular VRC amount is reversed by the inhibition of PKR or ISR.

**Figure 4:**
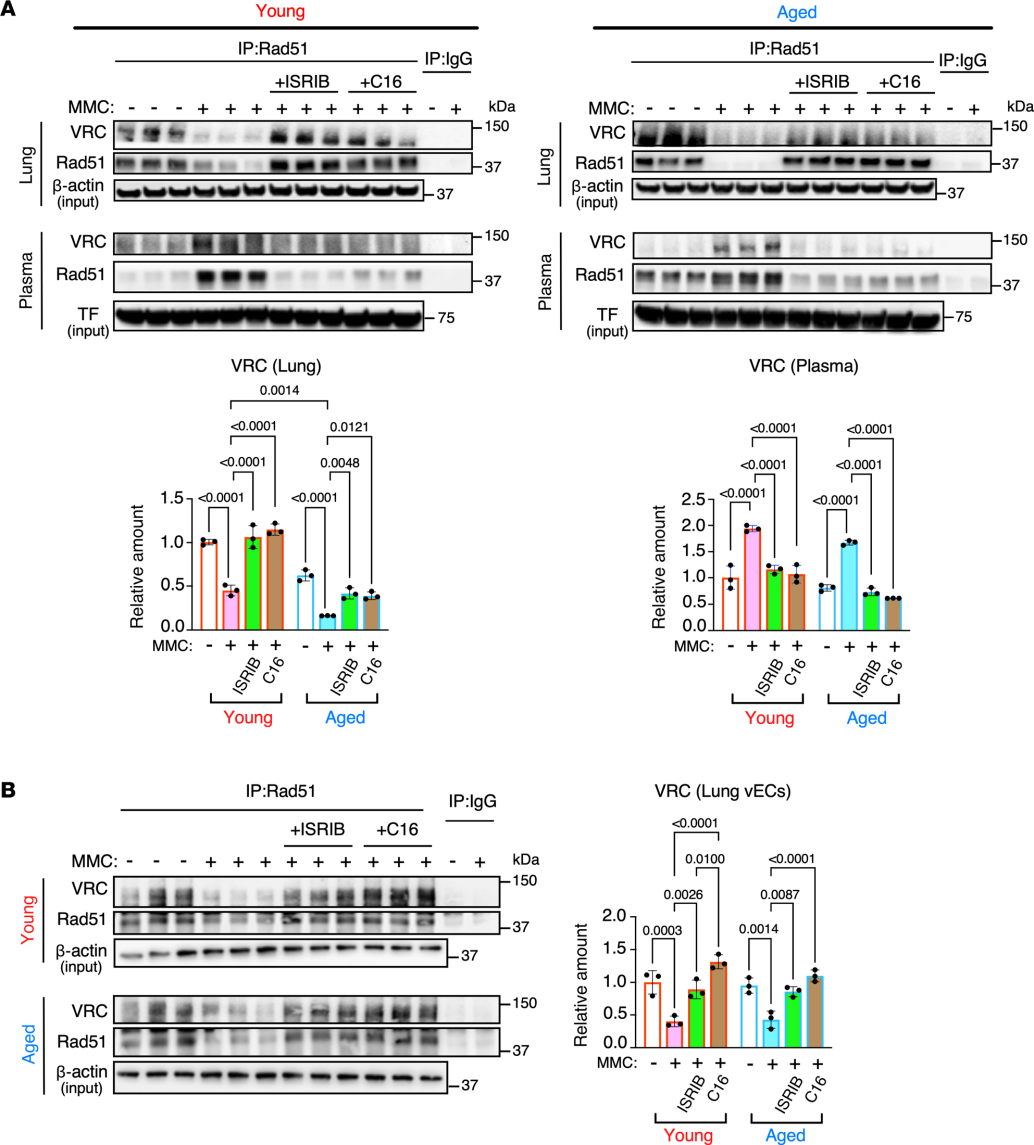
Inhibition the PKR/ISR axis blocks the release of VRC into the circulation. (**A**) Total lung lysates and plasma samples from vehicle (–), MMC (+), MMC+ISRIB, and MMC+C16 treated young and aged rats were subjected to immunoprecipitation (IP) by an anti-Rad51 antibody or nonspecific IgG (control), followed by immunoblot analysis of VE-Cad (for VRC) and Rad51. The total cell lysates and plasma samples without IP were subjected to immunoblot with anti–β-actin (for lung) and anti-transferrin (TF) antibody (for plasma) as loading control. The normalized amount of VRC in the lung and plasma are shown as mean ± SEM (bottom). *n* = 3 independent samples. The relative quantity of proteins was calculated by setting the amount of the protein in the vehicle-treated young rat to 1. (**B**) CD31^+^ pulmonary vECs were isolated from the lungs of vehicle (–), MMC (+), MMC+ISRIB, and MMC+C16 treated young and aged rats and subjected to the IP by an anti-Rad51 antibody or nonspecific IgG (control), followed by immunoblot analysis of VE-Cad (for VRC) and Rad51. The total cell lysates and plasma samples without IP were subjected to immunoblot with anti–β-actin (for lung) and anti-TF antibody (for plasma) as loading control. The relative amount of VRC is shown as mean ± SEM. *n* = 3 independent samples. Statistical analysis was performed using 2-way ANOVA with Tukey’s multiple comparisons test with *P* < 0.05.

Upon MMC treatment in rats, the VRC at the AJ is released into the circulation, resulting in the depletion of VRC within the endothelium and impairing junctional structure and barrier function (12). In the lungs of MMC-treated young and aged rats, VRC levels decreased by 55% and 74%, respectively, compared with the basal levels (Figure 4A and Supplemental Figure 2). Conversely, VRC levels in the plasma increased 1.9-fold and 2.1-fold in young and aged rats, respectively (Figure 4A and Supplemental Figure 2). After treatment with C16 or ISRIB, VRC levels in the lung were restored to the basal levels. Moreover, the elevated plasma VRC levels were reduced to the basal levels (Figure 4A and Supplemental Figure 2). Similar to the findings in the lung (Figure 4A and Supplemental Figure 2), VRC levels in CD31^+^ pulmonary vascular cells isolated from the rat lung decreased after MMC treatment (Figure 4B). However, the VRC levels were restored to their basal states when the rats were treated with C16 or ISRIB (Figure 4B). These results indicate that blocking the PKR/ISR axis restores endothelial adhesion proteins by preventing their release into circulation and mitigates endothelial injury and PVOD phenotypes, irrespective of the animal’s age.

### The lack of GADD34 induction and constitutive ISR activation following MMC treatment.

**Figure 5:**
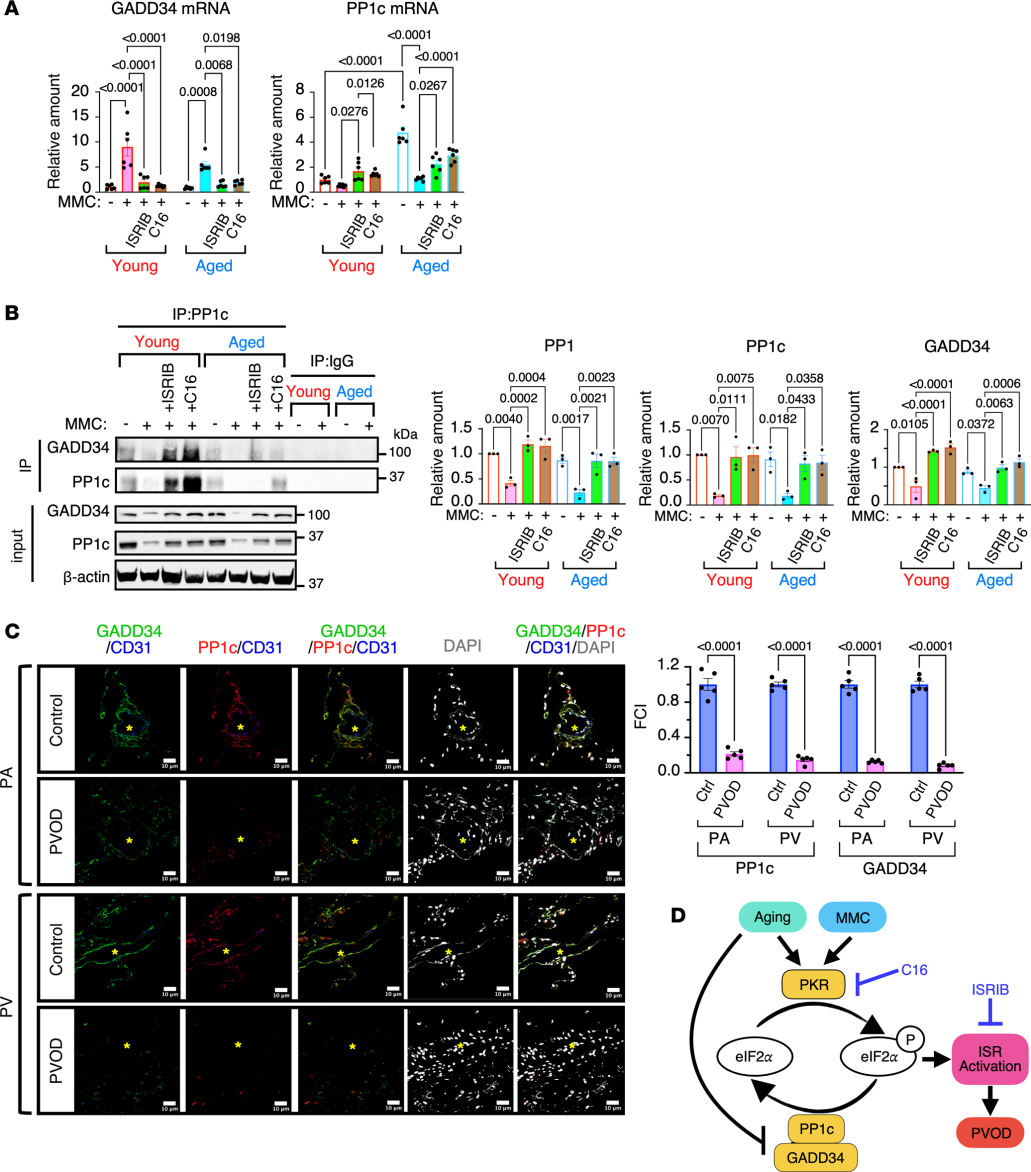
Reduced PP1 activity results in prolonged ISR activation. (**A**) The levels of GADD34 and PP1c mRNAs in the lung of young and aged rats treated with vehicle (–), MMC (+), MMC+ISRIB, and MMC+C16 were analyzed by qPCR, normalized to GAPDH, and shown as mean ± SEM. *n* = 6 independent samples. The relative quantity of mRNAs was calculated by setting the amount of the mRNA in the vehicle-treated young rat to 1. (**B**) The lung lysates of young and aged rats treated with vehicle (–), MMC (+), MMC+ISRIB, and MMC+C16 were subjected to immunoprecipitation (IP) with an anti-PP1c antibody or nonspecific IgG (control), followed by immunoblot with anti-GADD34 antibody to assess the amount of PP1 (the PP1c:GADD34 complex). Input samples were subjected to immunoblot analysis with PP1c, GADD34, and β-actin antibody (loading control). The amount of GADD34 in the IP samples (PP1) and the amount of PP1c and GADD34 in input samples were normalized to β-actin and shown as mean ± SEM. *n* = 3 independent samples. (**C**) The human lung samples from control individuals (Ctrl) and patients with IPAH were subjected to IF staining with anti-PP1c, anti-GADD34, and anti-CD31 antibodies, and the images of PAs and PVs are shown. Cell nuclei were stained with DAPI. The asterisk indicates the location of the vessels. Scale bar: 10 μm. The fluorescence signal intensities (FCI) of PP1c and GADD34 in the CD31^+^ cells in PAs and PVs were quantitated and plotted as mean ± SEM (right). *n* = 5 independent samples. (**D**) The schematic illustrates the pathway of constitutive ISR activation associated with aging, which exacerbates PVOD phenotypes. Age-related reduction in PP1 hinders the dephosphorylation of eIF2α, leading to global translational inhibition within vECs. This inhibition results in endothelial dysfunction and pulmonary vascular remodeling. Antagonists of PKR (C16) and ISR (ISRIB) can block prolonged ISR activation and reverse PVOD phenotypes. Statistical analysis was performed using 1-way ANOVA (**B**) and 2-way ANOVA with Tukey’s multiple comparisons test (**A** and **C**) with *P* < 0.05.

Dephosphorylation of eIF2α by PP1 (the PP1c:GADD34 complex) is critical in terminating the ISR signals and restoring protein synthesis after cellular stress (14, 17). Therefore, we assessed the PP1c (Ppp1cc) and GADD34 (Ppp1r15a) mRNA levels and protein in young and aged rats. The basal levels of GADD34 mRNA were similar between young and aged rats; the basal PP1c mRNA levels in aged rats were 4.8-fold higher than in young rats (Figure 5A). After MMC treatment, the GADD34 mRNA levels increased by 9.0-fold in young rats and 5.9-fold in aged rats (Figure 5A). This is due to the transcriptional activation of the GADD34 gene by ATF4 (18, 19). In contrast, the PP1c mRNA levels were decreased by 48% and 77% in young and aged rats, respectively, after MMC treatment (Figure 5A). Both the GADD34 and PP1c mRNA levels returned to the baseline (vehicle-treated rats) when rats were treated with ISRIB or C16 following MMC treatment (Figure 5A). Despite a robust increase in the GADD34 mRNA levels upon MMC treatment, the GADD34 protein amount decreased by 51% and 49% in young and aged rats, respectively (Figure 5B). Furthermore, PP1c protein amount decreased by 81% and 80% in young and aged rats, respectively, after MMC treatment (Figure 5B). Consequently, the amount of PP1 in MMC-treated young and aged rats reduced to 41% and 26% of vehicle-treated rats, respectively (Figure 5B). The reduction of PP1 by MMC was reversed upon ISRIB or C16 treatment (Figure 5B). The IF staining demonstrated the levels of PP1c and GADD34 were 80%–90% lower in CD31^+^ vECs in PAs and PVs of patients with PVOD compared with control individuals (Figure 5C). These findings, combined with previous results (12), suggest that the depletion of PP1 and persistent eIF2α phosphorylation following MMC treatment mediate maladaptive ISR responses, triggering PVOD pathogenesis in both rats and humans (Figure 5D). C16 or ISRIB treatment restores PP1, dephosphorylates eIF2α, terminates maladaptive ISR signals in the pulmonary vascular endothelium, and mitigates PVOD phenotypes in rats. Therefore, antagonists of the PKR/ISR axis hold the potential for treating patients with PVOD (Figure 5D).

## Discussion

Here, we demonstrate that the basal levels of ISR activity in aged rats are higher than in young rats, and the MMC-mediated PVOD phenotypes in aged rats are more severe than in young rats. PVOD affects both females and males across all age groups, though sporadic PVOD cases predominantly occur in males 60–70 years old (26). Several studies reported age-related changes in the levels of the eIF2 kinases in rodents (27). Increased levels of phosphorylated eIF2α have been detected in various organs from aged animals (27, 28). High levels of PKR have been detected in all tested tissues, including the lung and heart, in 20-month-old mice but not in 2-month-old mice (28). Muscle biopsies from human donors aged 20–80 also show increasing PKR abundance with age (28, 29). Thus, we speculate that the exposure to multiple stresses over time and the age-associated increase in the amount of PKR result in robust activation of ISR, leading to the sustained suppression of protein synthesis and VE damage and contributing to the higher incidence of PVOD among older individuals. Future studies using older rats, aged 2–2.5 years (equivalent to 60–75 years in humans; ref. 21), could provide further insights into the age-associated pathogenesis of PVOD.

We found that the antagonist of PKR or ISR effectively reverses PVOD phenotypes in both young and aged rats, even when the antagonists were administered after the PVOD phenotypes had fully developed, which further confirms that inhibiting the PKR/ISR axis might be an effective treatment even for the advanced stage of PVOD. Our findings demonstrate that the diminished levels of PP1c, coupled with the inability to induce GADD34 in the pulmonary vascular endothelium after MMC treatment, lead to the persistent activation of ISR signals. This results in a prolonged inhibition of protein synthesis. Consequently, what begins as a transient, adaptive ISR signal transforms into a constitutive, maladaptive ISR signal, leading to pathological remodeling of the pulmonary vasculature. PP1 is critical for regulating cognitive functions, such as learning and memory (30), synaptic transmission, and plasticity (31). The decline in memory associated with aging has been attributed to the depletion of the PP1c (32). Therefore, the reduction in PP1 activity underlies the pathogenesis of PVOD and age-associated cognitive decline. Our study highlights the therapeutic potential of restoring PP1 activity and attenuating maladaptive ISR signals to ameliorate PVOD and potentially mitigate cognitive deficits with aging.

## Methods

### Sex as a biological variable

Sex was not considered a biological variable in this study. Both male and female animals were used in all experiments.

### MMC-mediated PVOD rat model and administration of C16 and ISRIB

### Reagents, kits, antibodies, PCR primers, siRNAs, instruments, and software used in the study are listed in Supplemental Tables 1–4.

Sprague-Dawley rats were housed in the vivarium of the cardiovascular research building at UCSF. Both male and female young rats (9–10 weeks) and aged rats (1.2–1.5 years old) were subjected to the following protocols to examine the effect of MMC and/or small molecule inhibitors of ISR: ISRIB (IC_50_ = 5 nM; Sigma-Aldrich, 19785) or PKR antagonist: C16 (IC_50_ = 210 nM; Sigma-Aldrich, SML0843). The dosage of C16 and ISRIB was determined based on published studies in rodents (12, 33–37). According to the protocol, rats experienced no adverse effects. While rats treated only with the vehicle experienced a 12% weight gain after 24 days, those treated with MMC experienced no weight gain (12). When rats were treated with C16 or ISRIB following MMC treatment, they exhibited average weight gain similar to vehicle-treated rats (12). MMC was made by dissolving 2 mg MMC in 1 mL saline and was delivered to rats at 3 mg/kg dosage through i.p. injections. Saline was used as a vehicle solution for MMC treatment. ISRIB solution was made by dissolving 5 mg ISRIB in 1 mL of dimethyl sulfoxide (DMSO) (MilliporeSigma, D2650), followed by dilution to 1 mg/mL, and was delivered to rats at 0.25 mg/kg dosage through i.p. injections. The vehicle solution consisted of 1 mL DMSO and 4 mL saline. C16 solution was made by dissolving 10 mg C16 in 1 mL DMSO, followed by dilution to a final concentration of 100 mg/mL, and was delivered to rats at 33.5 mg/kg dosage through i.p. injections. The vehicle solution consisted of 100 mL DMSO and 10 mL saline.

#### Protocol no.1 MMC treatment.

Rats were randomly divided into groups exposed to MMC (3 mg/kg) or saline (vehicle). MMC or saline was administered once i.p. on day 0 (d0). Rats were euthanized on d24 for hemodynamic measurements, RV hypertrophy assessment, and tissue collections.

#### Protocol no.2 ISRIB/C16 treatment.

Rats were given MMC (3 mg/kg) or vehicle (saline) by i.p. on d0. ISRIB (0.25 mg/kg) or vehicle (DMSO) was given 3 times between d24 and d32, and rats were euthanized on d32. C16 (33.5 mg/kg) or vehicle (DMSO) was given once on d24. On d32, rats were euthanized for hemodynamic measurements, RV hypertrophy assessment, and tissue collections.

### Hemodynamic measurement and tissue histology

The animals were anesthetized with an i.p. injection of a ketamine/xylazine cocktail solution (1 mL ketamine [100 mg/mL] + 100 μL xylazine [20 mg/mL]; inject 300 μL per 250 g body weight). A tracheal cannula was then inserted, and the animals were ventilated with room air using a VentElite rodent ventilator (Harvard Apparatus) set to maintain respiration at 90 breaths/min and tidal volume at 8 mL/kg body weight. The abdominal and thoracic cavity of the rat was opened carefully to avoid any blood loss, and a 2F pressure-volume catheter (SPR-838, Millar AD Instruments) was used for the RVSP measurements. The RVSP was measured while a consistently stabilized pressure wave was shown after the transducer was plugged into the RV apex. At the end of the experiments, the hearts and lungs were perfused with PBS for blood removal. Fulton index, or the weight ratio of the right ventricle divided by the sum of the left ventricle and septum (RV/[LV + S]), was measured and calculated to determine the extent of right ventricular hypertrophy. Lung, liver, and heart tissues were fixed in 10% formalin for 24 hours and then further processed for paraffin sectioning. The paraffinized lung tissue sections were used for H&E (38) and Gomori’s trichrome staining (39) according to the standard protocol. The images were acquired by Olympus BX51 microscope (Olympus), Ts2 microscope (Nikon), and Leica SPE confocal microscope. Total areas of fibrotic lesions within each section were quantified using a threshold intensity program from ImageJ (NIH).

### Assessment of vascular remodeling

To assess PA and vein muscularization, rat lung tissue sections (10 μm in thickness) were subjected to conventional H&E staining. The external and internal diameter of a minimum of 50 transversally cut vessels in tissue block ranging from 25 to 80 μm was measured by determining the distance between the lamina elastica externa and lumen in 2 perpendicular directions described previously (40). The vessels were subdivided based on their diameter (microvessels: <50 μm and medium-sized vessels: 50–80 μm), and the assessment of muscularization was performed using ImageJ in a blinded fashion by a single researcher to reduce operator variability, which was not aware of the group allocation of the samples being analyzed. The absolute value of the medial thickness was converted to the relative value by setting the medial thickness of vehicle-treated WT rats as 1. We also assessed the muscularization of PAs and veins by the degree of αSMA IF staining. The IF signal intensity was quantitated by ImageJ, and the result is presented as a relative signal intensity by setting the value of vehicle-treated rats as 1. Images were acquired using Ts2 microscopes (Nikon) and Leica SPE confocal microscope. Airways (bronchi and bronchioles) follow a branching pattern that mirrors the tree-like structure of the lung’s lobes and segments, while veins have a more variable course as they drain blood back to the heart. Airways generally have thicker walls compared with veins. When cut in cross-section, they are more likely to maintain a round or oval shape, while veins may appear more collapsed or irregular. These characteristics were applied to distinguish veins from airways.

### Immunoblot analysis

The rat tissue lysates were prepared in the lysis buffer (1% Triton X-100, 150 mM NaCl, 50 mM Tris-Cl [pH 7.5], 1 mM EDTA). The supernatants were collected, and total protein concentration was measured by NanoDrop 2000c (Thermo Fisher Scientific). Protein samples were denatured in SDS-sample buffer for 5 min at 95°C, loaded onto Mini-Protean TGXTM gels (Bio-Rad) in equal amounts, and subjected to electrophoresis. Nitrocellulose membrane (Genesee Scientific) was used to blot the gels, which were blocked with 5% nonfat milk or 3% BSA in 1× Tris-buffered saline with 0.1% tween-20 (1× TBST) for 1 hour at room temperature. The membranes were incubated at 4°C overnight with a primary antibody. Chemiluminescence signals were detected using SuperSignal West Dura extended duration substrate (Thermo Fisher Scientific) and imaged using an Odyssey Dlx Imaging System (LI-COR). Antibodies used for immunoblots are found in Supplemental Table 2. The quantity of each protein was normalized to the amount of a loading control protein. Subsequently, its relative quantity was calculated by setting the amount of the protein in the control (vehicle-treated) sample as 1.

### Immunoprecipitation assay

Rat tissue and plasma samples were lysed in IP buffer (1% Triton X-100, 150 mM NaCl, 50 mM Tris-Cl [pH 7.5], 1 mM EDTA) supplemented with protease inhibitors (1:100 dilution) and phosphatase inhibitor (1:100 dilution). Lysates were nutated for 30 minutes at 4°C, followed by centrifugation at 12,000*g* for 10 minutes, and supernatants were collected. One-tenth of the lysate was saved as an input sample for immunoblot. The lysate was incubated with indicated antibodies and anti-IgG (negative control) nutating overnight at 4°C, followed by the addition of Dynabeads Protein A/G and rocking for 4 hours at 4°C. The magnetic beads were precipitated and rinsed thrice with IP buffer for 5 minutes at 4°C. The washed elute was boiled at 95˚C for 8 minutes in a sample loading buffer and subjected to immunoblot along with input. For the input samples, the quantity of the indicated protein was initially normalized to the amount of a loading control protein, such as β-actin. Subsequently, its relative quantity was calculated by setting the amount of the protein in the vehicle-treated sample as 1. For the IP samples, the amount of the indicated protein in the MMC-treated sample was presented with the protein amount in the control (vehicle-treated) sample set as 1.

### quantitative PCR (qPCR)

Total RNA was isolated from Rat lung tissue and subjected to cDNA preparation by the reverse transcription reaction using an iScript cDNA Synthesis Kit (17088890, Bio-Rad). qPCR analysis was performed in triplicate using iQ SYBR Green Supermix (1708882, Bio-Rad). The relative expression values were determined by normalization to *GAPDH* transcript levels and calculated using the ΔΔCT method. qPCR primer sequences are found in Supplemental Table 3.

### IF staining

Anesthetized rats were flushed with 1× PBS, fixed in 4% paraformaldehyde (w/v), transferred to 1× PBS after 24 hours, and subjected to paraffin embedding. The right bronchus of flushed lungs was sutured, the left lung inflated with 1% low melt agarose for fixation and paraffin embedding, and the right lung split for snap freezing for protein and RNA studies. IF was performed using antibodies listed in Supplemental Table 2. For SMA and EC staining, sections were subjected to deparaffinization, antigen retrieval, and permeabilization, followed by blocking and primary antibody incubation overnight at 4°C. Alexa Fluor secondary antibodies (Invitrogen) were applied for 2 hours at room temperature. IF images were acquired using a confocal microscope (Leica SPE) or Eclipse Ts2 Inverted LED phase contrast microscope (Nikon) and analyzed using ImageJ. Antibodies used are found in Supplemental Table 4.

### Isolation of pulmonary vECs

CD31^+^ pulmonary vECs were isolated from the lung tissues by an autoMACS NEO cell sorter (Miltenyi Biotec), according to the manufacturer’s manual. Briefly, rat lungs were isolated and enzymatically digested as previously described (41). Digestions were stopped after 20 minutes, and cells were processed into a single-cell suspension on ice. Cells were stained with CD31 magnetic beads. vECs were positively enriched using the autoMACS NEO cell sorter, and the total cell lysates were subjected to IP-immunoblot analysis.

### Human lung samples

Human lung tissues from patients with PH and control individuals were collected by the Pulmonary Hypertension Breakthrough Initiative (PHBI) in the US (http://www.ipahresearch.org/). Lung tissue from patients with PVOD and IPAH was obtained at the time of explant for lung transplantation. Control lung tissue was obtained from unsuccessful organ donors enrolled in the PHBI as controls. Clinical data for PH patients have been published (42). The PHBI follows a standardized tissue-processing protocol for lung tissue processing, as detailed previously (42).

### Statistics

All numerical data are presented as mean ± SEM. Statistical analysis was performed using Microsoft Excel and GraphPad Prism 10. Data sets with 2 groups were subjected to 2-tailed Student’s *t* test, unpaired, equal variance, whereas comparison among 3 or more than 3 groups was made by ANOVA followed by Tukey’s post hoc corrections. ANOVA was applied to experiments with multiple parameters, one- or 2-way, as appropriate. Significance was analyzed using a post hoc Tukey test and indicated as *P* values where required. A *P* value less than 0.05 was considered significant.

### Study approval

All animal experiments were conducted in accordance with the guidelines of the IACUC at UCSF. The relevant protocol, AN200674-00, was approved by the IACUC on July 5, 2023. Human samples were collected from patients enrolled in the PHBI. The study was approved by the Colorado Multiple Institutional Review Board (COMIRB) under Protocol 08-0423 on April 18, 2008. The collection of lung tissue was also approved by the IRB at all participating lung transplant sites.

### Data availability

No data sets were generated or analyzed during this study. Values associated with the main manuscript and supplement material are found in the Supporting Data Values file.

## Author contributions

Designing research studies was contributed by AP and AH. Conducting experiments and acquiring data were contributed by AP, MW, RK, and PG. Analyzing data was contributed by AP, MW, RK, BBG, RMT, GL, and AH. Providing reagents was contributed by AG and RMT. Writing the manuscript was contributed by AP, RK, RMT, BBG, GL, and AH.

## Supplementary Material

Supplemental data

Supporting data values

## Version 1 09/13/2024

In-Press Preview

## Version 2 10/08/2024

Electronic publication
