# Supplementary material for: Mechanisms underlying age-associated exacerbation of pulmonary veno-occlusive disease
Source: JCI Insight. 2024 Sep 5;9(19):e181877. doi: 10.1172/jci.insight.181877 (PMC11466196; doi:10.1172/jci.insight.181877)
Supplement: Supplemental data [file jciinsight-9-181877-s130.pdf]

Supplemental Figure S1 Prabhakar et al. 2024

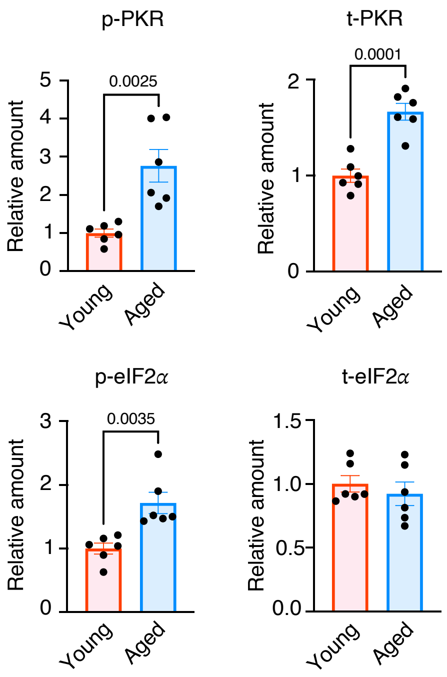

Supplemental Figure S1. Differential ISR activity in young and aged rats

Quantitation of immunoblot of p-PKR, t-PKR, p-eIF2α, and t-eIF2α in the lung lysates of young and aged rats shown in Fig. 1A. The amounts of indicated proteins normalized to β-actin are shown as mean±SEM (right). n=6 independent samples per condition. Statistical analysis was performed using a two-tailed Student's t-test with $p<0.05$.

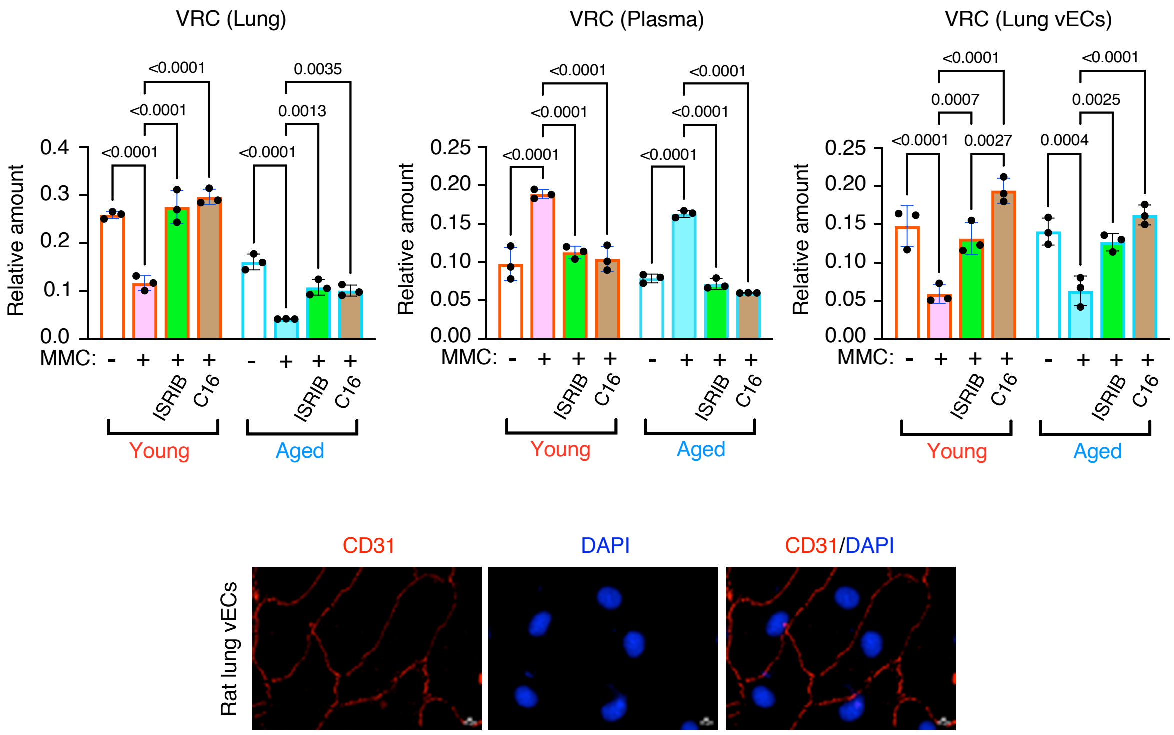

Supplemental Figure S2. Inhibition of the PKR-ISR axis attenuates the accumulation of VRC in the plasma and restores it in vascular endothelial cells. Top: Total lung lysates, plasma, and pulmonary endothelial cells (vECs) from vehicle (-), MMC (+), MMC+ISRIB, and MMC+C16 treated young (left) and aged rats (right) were subjected to immunoprecipitation (IP) by an anti-Rad51, followed by immunoblot analysis of VE-Cad (for VRC). The VRC amounts shown in Fig. 4A and 4B were quantitated and plotted without normalization as mean±SEM. n=3 independent samples. **Bottom:** vECs were subjected to Immunofluorescence staining for CD31 (red) and DAPI (blue). Scale bar=10 μm. Statistical analysis was performed using two-way ANOVA with Tukey's multiple comparisons test with $p<0.05$.

Supplemental Information

1. Reagents and kits

Reagent	Company	Catalog no.
C16	Sigma-Aldrich	I9785
DNase I	Ambion	AM2238
Dynabeads protein A	Invitrogen	10002D
Dynabeads protein G	Invitrogen	10004D
EGM-2 media	Lonza, Fisher scientific	CC-3162
Fetal Bovine Serum (FBS)	Fisher scientific	CC-4102B
ISRIB	Sigma-Aldrich	SML0843
Mitomycin C	Sigma-Aldrich	M0503
Mini-Protean TGX™ gels	Bio-Rad labs	
Nitrocellulose blotting membrane	Genesee Scientific	84-875
Protease Inhibitor	Sigma	P8340
Phosphatase Inhibitor	Sigma	P5726
RNase inhibitors	Invitrogen	AM2696
SuperSignal™ West Dura extended duration substrate	ThermoFisher	34076
SDS-PAGE sample buffer	Invitrogen	NP0007
SDS-PAGE reducing agent	Invitrogen	NP0009
Trypsin	Life technologies	25200-072
iScript cDNA synthesis kit	Bio-Rad labs	1708891
iQ SYBR Green supermix	Bio-Rad labs	1708885

2. Antibodies

Antigen	Company	Catalog no.
ATF4	Cell signaling Technology	11815
ATF4	Santa Cruz Biotechnology	sc-390063
α-smooth muscle actin	Sigma	A2547
α-smooth muscle actin	Novusbio	NB300-978
Alexa Flour 488 anti-rabbit IgG (H+L)	Life technologies	A21206
Alexa Flour 488 anti-mouse IgG (H+L)	Life technologies	A21202
Alexa Flour 555 anti-mouse IgG (H+L)	Life technologies	A32727
Alexa Flour 555 anti-goat IgG (H+L)	Life technologies	A21432
Alexa Flour 647 anti-mouse IgG (H+L)	Life technologies	A331571
β-actin	Sigma-Aldrich	A5441
eIF2α (total)	Cell signaling Technology	9722
eIF2α (total)	Santa Cruz Biotechnology	sc-133132
Phospho-Ser51-eIF2α	Cell signaling Technology	3597
GCN2	Cell signaling Technology	3302
GADD34	Proteintech	10449-1-AP

GADD34	Santa Cruz Biotechnology	sc-373815
PKR (total)	Proteintech	18244-1-AP
PKR (total)	Santa Cruz Biotechnology	sc-100378
Phospho-Thr446-PKR	Abcam	Ab32036
PP1c (E-9)	Santa Cruz Biotechnology	sc-7482
Rad51 (D4B10)	Cell signaling Technology	8875
Rad51	Abcam	Ab133534
VE-Cadherin	Cell signaling Technology	2500
VE-Cadherin	Santa Cruz Biotechnology	sc-9989
VE-Cadherin	Invitrogen	36-1900
Transferrin	Proteintech	17435-1-AP
PECAM1 (CD31)	Santa Cruz Biotechnology	Sc-376764
PECAM1 (CD31)	Invitrogen	PA5-143217
PECAM1 (CD31)	Miltenyi Biotec	130-126-036
IRDye-680RD goat anti-rabbit IgG (H+L)	Li-Cor	926-68071
IRDye-800CW goat anti-rabbit IgG (H+L)	Li-Cor	926-32211
IRDye-680RD goat anti-mouse IgG (H+L)	Li-Cor	926-68070
IRDye-800CW goat anti-mouse IgG (H+L)	Li-Cor	926-32210
anti-Rabbit-IgG-HRP-conjugated	Cell signaling Technology	7074
anti-Mouse-IgG-HRP- conjugated	Cell signaling Technology	7076

3. PCR Primers for RT-PCR and ChIP

**Primer Name**	**Primer Sequence**	**Annotation**
rAtf3-qPCR-F	5'-CCAGGTCTCTGCCTCAGAAG-3'	qRT-PCR for rat ATF3
rAtf3-qPCR-R	5'-AAGGTGCTTGTTCTGGATGG-3'	
rAtf4-qPCR-F	5'-TCCTCGATACCAGCAAATCC-3'	qRT-PCR for rat ATF4
rAtf4-qPCR-R	5'-ACCCATGAGGTTTGAAGTGC-3'	
rPkr-qPCR-F	5'-TTGCCTTCTGTGTTCTAGCCT-3'	qRT-PCR for rat PKR
rPkr-qPCR-R	5'-TCATCTGCCCACCCTGCTAT-3'	
rGadd34-qPCR-F	5'-ATGGAGTAAAGCAGCCCAGA-3'	qRT-PCR for rat GADD34
rGadd34-qPCR-R	5'-CTTGTCCTGGCTTCCCATTA-3'	
rPP1c-qPCR-F	5'-GGCCTATAAGATCAAATACCCGG-3'	qRT-PCR for rat PP1c

rPP1c-qPCR-R	5’-TGAAGCAGTCGGTGAAAGTC-3’	
rGapdh-qPCR-F	5’-TGGATAGGGTGGCCGAAGTA-3’	qRT-PCR for rat GAPDH
rGapdh-qPCR-R	5’-GGAAACCCTGCCATCCATCA-3’	

4. Instruments and softwares

Instrument/ software	Experiment	Company	Model no./ version no.
LI-COR	Immunoblot	Odyssey	Odyssey Dlx Imaging System
Dismembrator/Sonicator	Sonication	Fisher Scientific	550 sonic dismembrator
Tissue lyser	Tissue lysis	Qiagen	TissueLyser II
RT-PCR machine	qRT-PCR	BioRad	CFX connect
NanoDrop spectrometer	Protein, DNA, and RNA quantitation	Thermo Scientific	NanoDrop 2000c
Confocal Microscope	Immunofluorescence imaging	Leica	Leica SPE
Inverted Phase Contrast Microscope	Immunofluorescence imaging	Nikon	Eclipse TS2
Digital Color Microscope Camera	Attached to Eclipse TS2	Nikon	DS-Fi3
Rat Ventilator	RV catheterization	Harvard Apparatus	VentElite
2F Pressure-volume catheter	Hemodynamics measurement	Millar AD Instruments	SPR-838
Hemodynamics analysis software	Hemodynamics data analysis	AD Instruments	LabChart 8
MS Excel	Statistical analysis	Microsoft	MS office 365
GraphPad Prism	Statistical analysis	GraphPad	Prism 10
Cell sorter	Cell sorting	Miltenyi Biotec	autoMACS NEO
